# Application of Smart Mobile Medical Services in Maternal Health Care Management

**DOI:** 10.1155/2021/6249736

**Published:** 2021-12-08

**Authors:** Yue Liu, Xia Wang

**Affiliations:** ^1^Department of Nursing, Xuzhou Medical University, Huai'an Maternal and Child Health Care Hospital, Huai'an 221006, Xuzhou, China; ^2^Department of Nursing, Xuzhou Medical University, Xuzhou 221000, China

## Abstract

In order to standardize the health management of pregnant women, improve the health level of pregnant women, and improve the outcome of pregnancy with the help of the smartphone mobile terminal app, the 100 pregnant women who gave birth in the hospital and participated in the management of the health assistant app were selected as the observation group, and the 100 hospitalized pregnant women who did not participate in the management of the app were selected as the control group. The two groups of pregnant women were compared in their knowledge of health care, compliance of prenatal examination, delivery mode, and follow-up rate. The results showed that the observation group was significantly higher than the control group in the knowledge of health care during pregnancy and perinatal period, the rate of natural childbirth, the compliance rate of prenatal examination, and the follow-up rate. After the system was launched, the number of registered pregnant women reached more than 60% of the total number of pregnant women in the hospital, the number of clicks reached more than 2 million times, the number of clinic settlement accounted for more than 30%, and the interpretation rate of fetal heart rate in outpatient and remote clinics reached more than 20%. The diagnosis and treatment process has been significantly improved, and the implementation effect has reached the expectation. O2O maternal and child service mode has been realized through mobile internet technology. It has been proved that the use of smart mobile terminals in the out-of-hospital health care management of pregnant women not only facilitates medical staff to provide timely personalized medical services for pregnant women but also is convenient for pregnant women to obtain health care knowledge through multiple channels, improve the quality of home health management for pregnant women, and effectively improve the pregnancy outcome.

## 1. Introduction

With the development of economy and technology, people's requirements for their own health are gradually improving, and medical care and preventive medicine have been widely concerned; research conducted by the World Health Organization shows that effective preventive care and timely information communication play an important role in keeping people healthy [1]. However, the disadvantages of high cost and low efficiency of the traditional medical model become increasingly prominent, such as “difficult to see a doctor,” “three long and one short,” and other medical problems which have become the obstacles of people's health care. In this regard, China's medical and health system attaches great importance to the transformation of the medical model and puts forward the solution of medical informatization, strengthens the information construction of the medical and health system, and makes the majority of residents get complete health information records, convenient medical procedures, and high-quality medical and health services with the help of computer technology. Smartphones are the most common “personal computers” today, they have completely changed the communication environment, the rapid development of hardware makes them with rich display content and easy to operate the screen, more powerful computing power of the CPU, larger capacity of memory, and faster data transmission service network communication capabilities, these advantages make smart phones considered to be very promising health care tools, and the mobile health care model organically combined with smart terminal devices and mobile internet has become a new health service mode gradually recognized by people [2]. As one of the most important components of the public health service system, maternal and child health care institutions mainly serve young parents, which is very suitable for smartphone users; therefore, it is also a trend to gradually develop mobile maternal and child health care services [3]. The importance of pregnancy is self-evident for pregnant women, who are particularly eager to learn more about their health and to receive more guidance on maternal and child health care; this includes recording physical signs to monitor their health status in real time, inquiring about maternal and child health knowledge as needed, and convenient remote medical consultation and diagnosis. Meanwhile, the biggest advantages of smartphones are real-time communication, high portability, and rich software features; it is of practical importance to design and build an mHealth system that can improve maternal health care services based on these features [4].

With the implementation of the universal two-child policy, the number of pregnant women has increased significantly in recent years, popularizing scientific knowledge, improving the quality of maternal health management, and reducing the burden of hospital outpatient service which are the urgent problems to be solved in the health care work of maternal groups in China [5]. As an emerging means of socialized medicine, mobile medical platform has been gradually and widely applied in the medical service model [6]. Among them, mobile medical app has been accepted by the masses because of its simple and easy operation, the self-health management of pregnant women can effectively prevent pregnancy complications and fetal health and safety problems, and good health care during pregnancy and childbirth can improve the health of the mother and child. This study reviewed the application of maternal health management app in order to provide reference for similar studies in the future [7]. Therefore, the development of information technology has promoted the reform of human resource management. The application of intelligent attendance technology has changed the traditional way of attendance, improved the efficiency of human resource management, and provided better service for the organizational strategy. Wu et al. discussed new attendance methods, including face recognition, fingerprint recognition, iris recognition, and other nonmobile attendance, and enterprise WeChat, tripod attendance, and other mobile attendance, and the application of five different intelligent attendance technologies is compared and analyzed. It is proposed to improve mobile attendance app, deepen electronic human resource management, mine big data of human resource management, and realize the deep integration of intelligent attendance technology and human resource management in further research [1]. Andreev et al. designed an intelligent drug management platform based on deep learning. Based on the intelligent medicine box, combined with mobile app, WeChat official account, mini program, etc., the platform can not only realize the functions of timing, intelligent reminder, medication record feedback, etc, but also provide medication information and multiservice supervision for patients, which can significantly improve patients' irregular medication and reduce drug leakage [8]. Xu et al. studied the particular difficulty of emergency care due to the transient and dynamic individual patient environment; all methods currently implemented are designed regardless of physical states and different manifestations of cognitive impairment. Therefore, our goal is to design and integrate a telemedicine platform, which is an intelligent user interface solution that can be adapted to clinical emergency patient situations in real time. The present study proposes a mobile implementation of hypoglycemic therapy for diabetes. Continuous physiological data from medical-grade wearable sensors, as well as customized cognitive training results evaluating corresponding common emergency symptoms, are used to define patient status [9]. On the basis of current research, it is proposed to standardize the health management of pregnant women with the help of smartphone mobile terminal app, improve the health level of pregnant women, and improve the outcome of pregnancy. It has been proved that the use of smart mobile terminals in the out-of-hospital health care management of pregnant women not only facilitates medical staff to provide timely personalized medical services for pregnant women but also is convenient for pregnant women to obtain health care knowledge through multiple channels, improve the quality of home health management of pregnant women, and effectively improve the pregnancy outcome [10].

Mobile medical system constructed by mobile Internet can effectively remind patients with gestational diabetes and transmit data automatically. For mothers and nurses, communication has brought the convenience, but this approach in patients with gestational diabetes blood sugar control and neonatal birth weight on both primary endpoint events did not see a significant difference. Due to the increase of the diabetes population, the number of specialized health care teams for diabetes-related pregnancy is relatively reduced. However, the mHealth system can effectively reduce the number of patient visits and play a positive role in the rational allocation of health system resources. Both patients and the health care system can benefit from this. Although the results of the two studies are different, the positive effects of mHealth in this particular group of diabetics are worth looking forward to.

## 2. Research Objects and Methods

### 2.1. Research Objects

200 pregnant women who underwent routine prenatal examination in the outpatient department of a hospital and gave birth in the hospital from July 2019 to June 2020 were selected as the research subjects, the 100 pregnant women who were managed by the health assistant app were the observation group, and the other 100 cases who were not used were the control group. All the selected candidates were primiparas, aged 20–31 years, with an average age of 25.1 ± 3.38 years, and educational level as follows: 36 were junior college or above, 141 were technical secondary school or high school, and 23 were junior middle school or below. There was no significant statistical difference between the two groups in general information, such as age, gestational age, and educational background (*P* > 0.05).

### 2.2. Methods

#### 2.2.1. Methods Adopted in the Two Groups

The control group received routine prenatal examination, routine pregnancy education by outpatient nurses, routine perinatal health education by inpatient nurses after delivery, and one telephone follow-up visit after discharge. The observation group adopted health assistant app to implement multichannel health education management for pregnant women [5, 11].

#### 2.2.2. Health Assistant App Intervention Management

Health assistant app consists of 8 modules: department management, follow-up list, basic patient information, discharge record (outpatient medical record), follow-up method, follow-up content, follow-up evaluation, and follow-up appointment; the whole process of follow-up task management covering prenatal examination, postpartum visit, and newborn visit has been established. By connecting with the HIS, the health assistant app can automatically read related information of pregnant women from the electronic medical record, automatically screen and classify tasks according to the preset program, and intelligently remind medical staff to complete their follow-up tasks [12, 13].

#### 2.2.3. App Process

**Figure 1:**
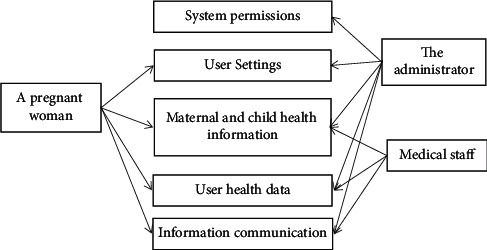
App analysis diagram.

① Use the encryption authentication mode. Enter the username and password to log in to the user account. ② After login, click the department to enter the page of follow-up list, to be followed up, followed up, and expired; the list page contains patient name, gender, and management physician information. Patient information can be quickly queried by name. Basic patient information includes name, gender, age, admission time, discharge time, diagnosis, contact number, occupation, and home address. ③ Select the follow-up object, click enter, and you can view the basic personal information of patients and outpatient records (discharge records). ④ Choose follow-up methods, such as telephone, information, receiving consultation service, and WeChat. One-click dialing, one-click information sending of health knowledge guidance and consultation notification, and WeChat template push can be adopted. ⑤ Administrators can view the follow-up of general practitioners, conduct follow-up investigation, and systematically evaluate the satisfaction (Figure 1) [14, 15].

#### 2.2.4. Smart Watches

Smart watches automatically record and upload daily sleep, exercise, and other health management indicators of pregnant women through mobile phones so that pregnant women can develop healthy habits of sleep and exercise before pregnancy. To improve the success rate of conception, clothing thickness can be adjusted by body surface temperature, and ovulation time can be mastered by basic temperature change. This provides a 24-hour online management secretary for pregnant women, especially high-risk pregnant women, who can provide a reliable reference basis for postpartum rehabilitation under the guidance of postpartum health care, sleep, and exercise.

#### 2.2.5. Smart Fetus

Smart fetal heart device is a miniature Doppler tire with Bluetooth connection function. Equipped with a deseparated detection probe, the device connects the pregnant woman's mobile phone through Bluetooth to measure and record data. After recording, the pregnant woman can choose whether to upload the data to the cloud service center. After the data are uploaded, pregnant women can choose whether to interpret them or not. When the pregnant woman chooses to be interpreted, the doctor at the interpretation center will interpret the record and give opinions on the interpretation.

### 2.3. Effect Evaluation

The observation group was compared with the control group in terms of prenatal examination times, pregnancy health care cognitive behavior, delivery mode, follow-up times, and satisfaction evaluation, and the two groups of women were evaluated by questionnaire, clinical data review, and health assistant app automatic statistics to explore the effect of the application of the health assistant app on improving maternal health management quality and pregnancy outcome [16, 17].

### 2.4. Statistical Methods

SPSS 17.0 statistical software was used for data analysis, and *x*^2^ test was used for comparison between groups.

## 3. Results

### 3.1. Comparison of Cognitive Behavior of Maternal Care during Pregnancy and Perinatal Period between the Two Groups

The observation group mastered the cognitive behavior of health care during pregnancy and perinatal period better than the control group, with a statistically significant difference (*P* < 0.01), as shown in Table 1.

### 3.2. Comparison of Prenatal Examination Times and Delivery Modes between the Two Groups

The frequency of prenatal examination and the number of cases of natural delivery in the observation group were higher than those in the control group, with statistically significant differences (*P* < 0.01), as shown in Table 2.

### 3.3. Comparison of the Follow-Up Rate and Satisfaction of Patients in the Two Groups

In the observation group, the rate of prenatal follow-up ≥3 times was 95.6%, the rate of postpartum follow-up ≥2 times was 100%, and the patient satisfaction was 100%. In the control group, the rate of prenatal follow-up ≥3 times was 18.5%, the rate of postpartum follow-up ≥2 times was 28%, and the patient satisfaction was 92.8%. The statistical difference was significant (*P* < 0.01).

## 4. Discussion

### 4.1. Complete Interconnection of Pregnancy Health Information

At present, most maternity management systems only connect to midwifery units and have no connection to maternity records or HIS. These systems are very limited and not interconnected. Through the development of the health assistant app, three interconnections have been realized: one is the seamless connection with personal electronic health records, with ID number as the unique identification number; an ID card has only one electronic file in the hospital system to avoid the error of manual statistics. The second is to realize the interconnection of pregnancy information and antenatal information; through the combination of patient ID and diagnosis, the data of pregnant women can be automatically entered into the files of pregnant women, ensuring the convenient management of the whole file of pregnancy and childbirth [18]. The third is the establishment of child health information management; through the mother ID, the child's birth information and postpartum follow-up information are automatically imported, so as to realize a set of continuous and complete health files during pregnancy, perinatal period, and neonatal period, and make the pregnancy and childbirth management more scientific, standardized, effective, and feasible. Maternal and child health hospitals mainly cater to women and children, with particular emphasis on pregnancy services. Pregnant women are particularly problematic during pregnancy. In order to meet the needs of pregnant women in terms of health, medical treatment, and health care, pregnant women can consult obstetricians through the app. Through the personal signs uploaded by pregnant women, doctors can learn about the physical signs of pregnant women remotely; at the same time, they can access the personal pregnancy health record system to check the personal birth examination records of pregnant women and put forward targeted guidance and suggestions. Pregnant women do not have to personally run to the hospital; regular problems can be solved by online counseling.

The system automatically collects, stores, and uses statistical information, associating new information with past information and showing the current health status of pregnant women in a timely and comprehensive manner. The complete storage of health records of pregnant women and women can be realized to provide accurate and fast data for medical staff, so as to facilitate medical staff to carry out personalized health education work. Through the system design, the function of appointment inspection, and automatic reminder of follow-up expiration, it is convenient for medical staff to carry out health guidance and remind pregnant women to carry out pregnancy examination in time, thus improving the quality of maternal health management. The app is simple and convenient to use. You can access the internet through your mobile phone and log in directly, which solves the problem of discontinuous information under the traditional health care mode. At the same time, the health assistant app is deployed on two servers to ensure the security and stability of the system through comprehensive use of data encryption technology and management methods.

### 4.2. Platform Functions Are Easily Accepted by Doctors and Patients

**Figure 2:**
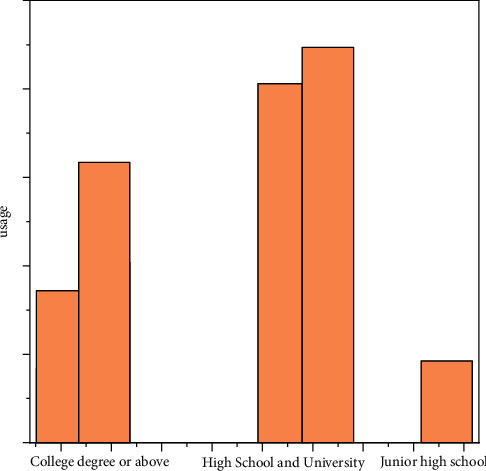
Usage comparison.

As can be seen from Figure 2, the utilization rate of high school and college degrees is relatively higher than that of junior college and junior high school. Health assistant app can intelligently remind medical staff to promote maternal health behaviors in multiple forms and multidimensions; it not only facilitates medical treatment but also improves the compliance rate of prenatal examination and health knowledge of pregnant women. In addition, through the expansion of WeChat public platform functions, one is to achieve advance appointment and shorten the time to see a doctor; second, push health education materials in the form of pictures and videos on a regular basis to make the education more intuitive and easy to understand. At the same time, pregnant women should obtain appropriate health information according to their own needs, so as to achieve a targeted goal and avoid one-time indoctrination which becomes a formality. The results of this study indicate that the timely follow-up of antenatal clinic can not only improve the regular antenatal examination and timely detection of birth defects but also increase the maternal compliance with pregnancy health care. Health assistant app provides a convenient, personalized, fast, effective, and spatial-temporal service mode for continuous service, which is worthy of clinical application and promotion. Mobile internet technology can enhance traditional forms of television, radio, and live education. Through live broadcasting and retrospecting, the curriculum of the maternity school can be put online. Pregnant women can inquire about health knowledge and watch health education courses anytime and anywhere with just a few taps on their mobile phones, forming an effective supplement to traditional health education methods.

### 4.3. Shortcomings

The emergence of mobile health apps brings challenges as well as opportunities for the development of maternal health. With the increasing use of apps for pregnant women, people are paying much attention to the quality of their content, the authenticity of online resources, and the security of personal information. The extensive use of mobile health involves a large number of health data, the leakage will bring serious adverse impact on the development of the mobile health industry, personal information security, and other issues, but China's mobile health app field supervision and legal norms are almost blank. At present, the mobile medical app for pregnancy health care has the following problems: low threshold, market chaos, lack of professionalism in the information content, lack of standard diagnosis and treatment procedures and use guidelines for mHealth, relative lack of technical personnel, etc. Therefore, China needs to formulate laws and regulations related to the mobile medical app as soon as possible to make the mobile medical market more standardized; at the same time, we are constantly optimizing the app, improve user experience, and better play its social benefits for maternal health and life to bring better experience.

## 5. Conclusions

In order to standardize the health management of pregnant women, improve the health level of pregnant women, and improve the outcome of pregnancy with the help of the smartphone mobile terminal app, the 100 pregnant women who gave birth in the hospital and participated in the management of the health assistant app were selected as the observation group, and the 100 hospitalized pregnant women who did not participate in the management of the app were selected as the control group. The two groups of pregnant women were compared in their knowledge of health care, compliance of prenatal examination, delivery mode, and follow-up rate. The results showed that the observation group was significantly higher than the control group in the knowledge of health care during pregnancy and perinatal period, the rate of natural childbirth, the compliance rate of prenatal examination, and the follow-up rate. It has been proved that the use of smart mobile terminals in the out-of-hospital health care management of pregnant women not only facilitates medical staff to provide timely personalized medical services for pregnant women but also is convenient for pregnant women to obtain health care knowledge through multiple channels, improve the quality of home health management for pregnant women, and effectively improve the pregnancy outcome.

## Figures and Tables

**Table 1:** Comparison of cognitive behavior between the two groups.

Group	*n*	Maturity is nutrition and self-monitoring during pregnancy	Familiar with the labor process and labor symptoms	Familiarize yourself with puerperal self-care	Understand the importance and methods of breastfeeding	Know how to care for newborns
Observation group	100	84	83	79	89	75
Control group	100	42	34	42	41	28
*X* ^2^		63%	58.5%	60.5%	65%	51.5%
*P*		<0.01	<0.01	<0.01	<0.01	<0.01

**Table 2:** Comparison of the number of obstetric examinations and pregnancy outcomes between the two groups (*n* = 100).

Project	Number of antenatal examinations		Delivery way		Cesarean section indicators		Complications of pregnancy and childbirth
	≥5 times	<5 times	Natural birth	Cesarean delivery	Social factors	Medical indicators	
Observation group	96	4	67 (67.00)	13 (13.00)	2	11	1
Control group	57	43	48 (48.00)	32 (32.00)	14	18	3
*X* ^2^	76.5%	23.5%	57.5%	22.5%	8.0%	14.5%	2%
*P*	<0.01	<0.01	<0.01	<0.01	<0.01	<0.01	<0.01

## Data Availability

The data used to support the findings of this study are available from the corresponding author upon request.
